# Conservative treatment for brachial plexus injury after a displaced clavicle fracture: a case report and literature review

**DOI:** 10.1186/s12891-022-05601-5

**Published:** 2022-07-02

**Authors:** Myung-Seo Kim

**Affiliations:** Shoulder & Elbow Clinic, Department of Orthopaedic Surgery, College of Medicine, Kyung Hee University Hospital at Gangdong, 892, Dongnam-ro, Gangdong-gu, 05278 Seoul, Republic of Korea

**Keywords:** Clavicle fracture, Brachial plexus injury, Conservative treatment, Clinical outcome, Case report

## Abstract

**Background:**

A brachial plexus injury is a rare complication of clavicle fractures. Previous case reports only examined the surgical outcomes of brachial plexus injuries that occurred after a clavicle fracture and did not report on the outcomes of conservative treatment. In this report, we present the prognosis of a patient with an acute displaced clavicle fracture accompanied by a brachial plexus injury that was conservatively treated.

**Case presentation:**

A 51-year-old man with a middle-third clavicle fracture due to a direct trauma after falling down the stairs. A brachial plexus injury experienced symptoms, including numbness occurred in the affected upper extremity, at 1 day after the injury. The patient’s motor power in the elbow, wrist, and hand decreased at 3 days after the injury. Magnetic resonance imaging (MRI) showed no loss of continuity in the brachial plexus, but showed nerve compression by displaced fracture fragments. Electromyography revealed brachial plexopathy. Conservative treatment, including a shoulder sling, was performed with satisfactory outcomes; the patient reported a 70% improvement at 6 months after the injury.

**Conclusions:**

A brachial plexus injury is a rare complication of clavicle fractures that can cause serious dysfunction of the upper extremities affected by the injury. Conservative treatment may be considered for acute nerve compression by displaced fracture fragments rather than extensive callus or granulation tissue formation to achieve a satisfactory recovery in young patients. MRI should typically be performed before making a treatment decision to examine the brachial plexus for any discontinuity or kinking.

## Background

A brachial plexus injury is a rare complication of clavicle fractures. In addition, there have only been a few case reports on brachial plexus injury accompanying clavicle fractures [1–3]. Saito et al. retrospectively analyzed the data collected from patients with clavicle fractures from 2004 to 2018 and reported that only three patients had brachial plexus palsy [4]. Barbier et al. reported satisfactory outcomes after performing open reduction and internal fixation (ORIF) and neurolysis on patients whose brachial plexuses were compressed by the displaced bone fragments [5]. Moreover, Gadinsky et al. described in their case report that surgery successfully resolved the neurologic symptoms [2]. However, these studies only examined the surgical outcomes of brachial plexus injuries that occurred after a clavicle fracture and did not report on the outcomes of conservative treatment. In this report, we present the prognosis of a patient with an acute displaced clavicle fracture accompanied by a brachial plexus injury that was conservatively treated.

### Case presentation

**Fig. 1:**
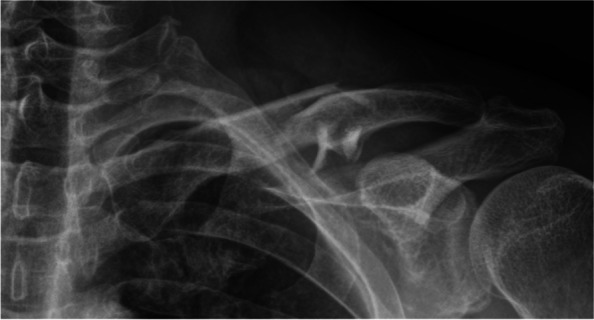
Comminuted midshaft left clavicle fracture (Robinson classification type 2B2)

The patient was a 51-year-old man with a displaced middle-third left clavicle fracture (Robinson classification type 2B2) (Fig. 1). The patient, who had diabetes mellitus and hypertension as comorbidities, experienced a direct trauma after falling down the stairs. Despite the patient’s history of consistent aspirin use, his prothrombin time and international normalized ratio was 1.0 during a laboratory test, indicating no prolongation.

**Fig. 2:**
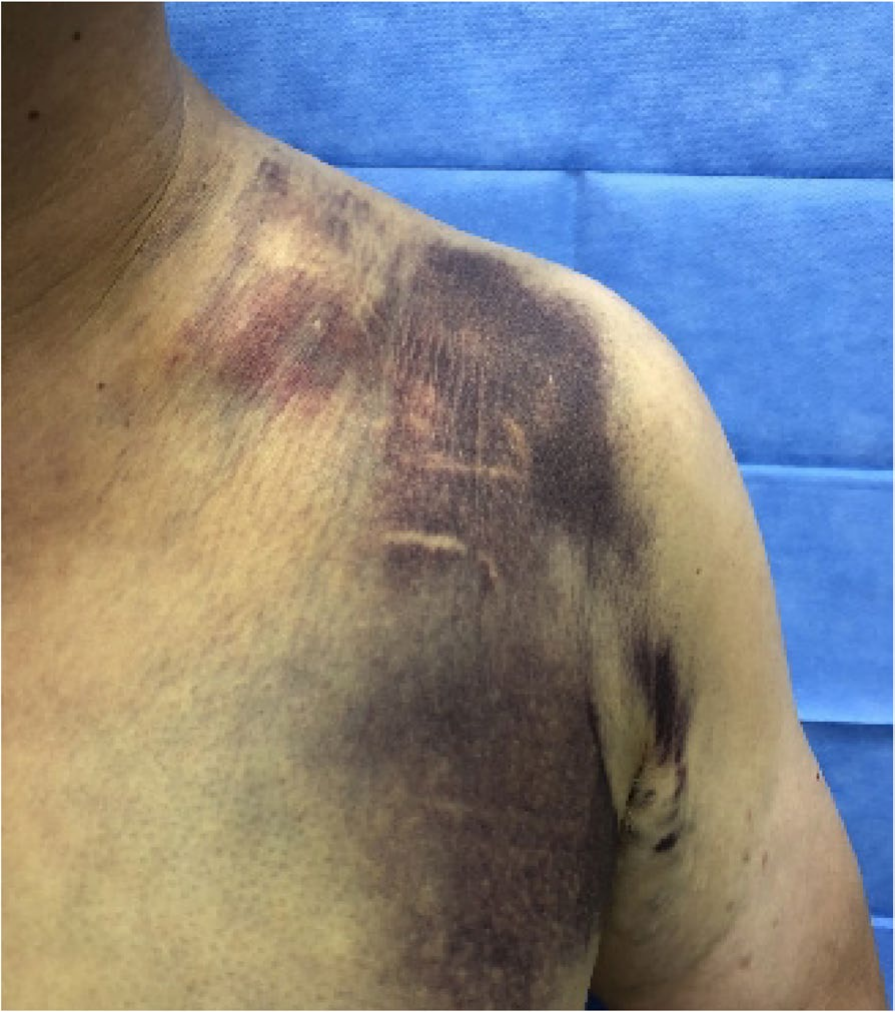
Severe bruising around the fracture site

The patient did not receive any treatment immediately after the injury. However, at 1 day after the injury, the patient was prescribed with a shoulder sling and underwent conservative treatment at an outpatient local clinic due to left upper extremity. At 3 days after the injury, the patient was admitted to our institution with chief complaints of worsened hand numbness and immobility. The patient had a severe bruise around the site of the clavicle fracture at the time of admission. Skin tenting caused by displaced fracture fragments was not observed (Fig. 2). A manual muscle strength test was performed using the Medical Research Council (MRC) scale [6], showed grades 2, 0, 3, 0, and 0 on elbow flexion, elbow extension, wrist flexion, wrist extension, and thumb and other finger extension, respectively; shoulder abduction was not examined since the patient had a clavicle fracture. The pulse could be palpated over the brachial artery at the elbow level and over the radial and ulnar arteries at the wrist level. Furthermore, the distal phalanx of the left hand showed an intact capillary refill.

**Fig. 3:**
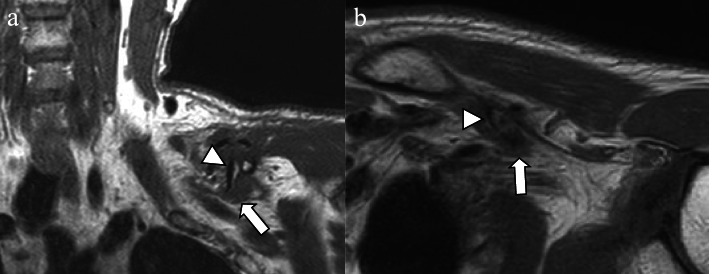
Magnetic resonance imaging **(a)** T1-weighted coronal; (**b)** axial brachial plexus magnetic resonance imaging: brachial plexus (arrow) was compressed by the displaced comminuted fracture fragment (arrowheads)

No cervical spine injury-related abnormalities were observed. In magnetic resonance imaging (MRI) performed 3 days after the injury, brachial plexus discontinuity was not observed in the T1-weighted coronal and axial view, although the brachial plexus was compressed by the displaced midshaft fracture fragments (Fig. 3). MRI was performed with the patient in the supine position using a Philips Ingenia 3.0-T scanner (Philips Medical Systems, Best, Amsterdam, Netherlands) with a dedicated anterior and head/neck 16-channel coil. A detailed MRI protocol was added as Table 1.

**Table 1:** Magnetic resonance imaging protocol

Sequence	T1-weighted spin echo		CE T1-weighted MDIXON spin echo	
	Axial	Coronal	Axial	Coronal
Repetition time, ms	488	639	625	568
Echo time, ms	15	15	15	15
Slice thickness, mm	3	2	3	2
Slice gap, mm	0	0	0	0
Field of view, mm	250 × 250	250 × 250	250 × 250	250 × 250
Matrix size	320 × 257	284 × 218	320 × 248	356 × 232
Echo train length	4	6	6	7

*CE* Contrast enhanced, *MDIXON* Modified DIXON

Electromyography (EMG) performed 3 weeks after the injury revealed brachial plexopathy at the whole trunk level.

Conservative treatment, including a shoulder sling, was performed for 4 weeks at our institution. At 4 weeks after the injury, active-assisted motion exercised were started when the shoulder sling was weaned. At 11 days after the injury, both the wrist extension and extension power of the thumb and other fingers improved from MRC grade 0 to 1. No significant improvements in motor power were observed at 27 days after the injury, although the patient reported of more arm strength during the subjective assessment. The follow-up EMG was scheduled at 3 months after the injury to assess the extent of recovery from the brachial plexus injury. However, the patient refused EMG, and since an examination at an outpatient clinic was difficult owing to the patient’s personal reasons, a follow-up telephone interview by audio reporting was conducted instead. At approximately 6 months after the injury, we assessed the patient’s recovery status through a telephone interview. The patient could perform shoulder abduction up to 90° without experiencing pain, and the muscle strength was improved to grade 4. Elbow flexion and extension were improved to MRC grade 5 (the patient reported being able to lift up to 20 kg with the affected arm) and wrist flexion and extension as well as flexion and extension of the thumb and other fingers improved to MRC grade 4. The numbness in the affected upper extremities subsided. The patient had an overall 70% improvement compared with his condition at the time of the injury, showing a satisfactory recovery.

## Discussion and conclusions

Clavicle fractures account for approximately 3% of all fractures, with midshaft clavicle fractures reported as the most common type [7, 8]. Conservative treatment has been commonly performed for midshaft clavicle fractures with satisfactory outcomes in several studies [9, 10]. However, conservative treatment is reported to have higher rates of nonunion and malunion than surgical treatment [11, 12], which may result in neurologic injuries in rare cases. Thavarajah et al. reported that extensive callus resulting from the hypertrophic nonunion can compress the brachial plexus [13]. Gadinsky et al. also reported two cases of brachial plexus compression by abundant calluses at 4 months and 5 weeks after the conservative treatment of displaced clavicle fractures, respectively [2].

Although the abnormal callus or granulation tissue formation is the most common cause of brachial plexus injuries and neurologic symptoms often manifest several weeks to months after the injury [14], Nordqvist et al. reported that 2% of the displaced clavicle fractures are accompanied by primary brachial plexus injuries [15]. Lin et al. reported a case of brachial plexus compression by a displaced fragment, although the direct compression of the brachial plexus by a displaced fragment in an acute clavicle facture is rare [3]. Della Santa et al. stated that of the 16 brachial plexus lesion cases after a clavicle fracture that were examined over 20 years, only a single case showed direct compression of the brachial plexus by a displaced fracture fragment [16]. In our case report, the patient had numbness in the affected upper extremity at 1 day after the injury; thus, he did not have a chronic brachial plexus injury. In addition, the patient showed direct compression of the brachial plexus by a displaced comminuted fracture fragment.

Saito et al. revealed that subacute brachial plexus palsy can gradually occur in older adult patients exhibiting abnormal growth of granulation tissues around the site of the middle-third clavicle fracture [4]. Gadinsky et al. reported two brachial plexus injury cases, one of which was a 71-year-old patient with a midshaft fracture, who experienced weakness in the left upper extremity at 5 weeks after the injury [2]. The patient in our case report was 51 years old and was thus relatively young, but had a middle-third clavicle fracture. The patient also had neurologic symptoms at 1 day after the injury, indicating that an acute brachial plexus injury can result from the direct compression of the brachial plexus by a bone fragment.

In previous studies, the extensive calluses and granulation tissues, which are the causes of brachial plexus compression, were removed for most subacute or chronic brachial plexus injury cases that occurred after a clavicle fracture, and resection or ORIF was performed for the clavicle fracture. They reported a complete muscle strength recovery as early as 3 months and usually within 6–8 months after surgery [2–4]. Our patient did not lose nerve continuity as a result of the acute brachial plexus injury accompanying a clavicle fracture, and the conservative treatment was performed since no kinking was observed between fragments. The patient showed a satisfactory recovery of approximately 70% at 6 months after the injury. The patient was in employment before the injury and returned to work at the time of the telephone interview.

To our knowledge, several studies have reported satisfactory outcomes after performing decompression, neurolysis, and ORIF for brachial plexus injuries accompanied by a clavicle fracture. However, no study has reported on the outcomes of conservative treatment. In this study, since clinical outcomes at 3 months after the injury were evaluated by telephone interview through audio reporting, clinical data was insufficient and recall bias might have occurred**.** Nevertheless, this case report is meaningful in that a satisfactory prognosis was observed following the conservative treatment of an acute brachial plexus injury that occurred after a clavicle fracture. Further research using a larger number of cases and long-term follow-up data is needed to assess the prognosis of conservative treatment for a brachial plexus injury accompanying a clavicle fracture.

A brachial plexus injury is a rare complication of clavicle fractures that can cause serious dysfunction of the upper extremities affected by the injury. An appropriate treatment must be performed to recover from the nerve injury, and satisfactory prognoses can be expected from surgical procedures, such as ORIF and brachial plexus decompression. Conservative treatment may be considered for acute nerve compression by displaced fracture fragments on young patients rather than by extensive callus or granulation tissue formation as a management method to achieve a satisfactory recovery. MRI should typically be performed before making a treatment decision to examine the brachial plexus for any discontinuity or kinking.

## Data Availability

The authors declare that all data used during the study appear in the submitted article.

## Abbreviations

MRI: Magnetic resonance imaging
ORIF: Open reduction and internal fixation
EMG: Electromyography

## References

[1] Balfousias T, Apostolopoulos AP, Papanikolaou A, Karadimas E, Zouboulis G, Maris I (2018). Scapulothoracic Dissociation and Clavicle Fracture with Associated Brachial Plexus Palsy. J Long Term Eff Med Implants.

[2] Gadinsky NE, Smolev ET, Ricci MJ, Mintz DN, Wellman DS (2019). Two cases of brachial plexus compression secondary to displaced clavicle fractures. Trauma Case Rep.

[3] Lin CC, Lin J: Brachial plexus palsy caused by secondary fracture displacement in a patient with closed clavicle fracture. Orthopedics 2009;32(10). 10.3928/01477447-20090818-24 19824597

[4] Saito T, Matusmura T, Takeshita K (2020). Brachial plexus palsy after clavicle fracture: 3 cases. J Shoulder Elbow Surg.

[5] Barbier O, Malghem J, Delaere O, Vande Berg B, Rombouts JJ (1997). Injury to the brachial plexus by a fragment of bone after fracture of the clavicle. J Bone Joint Surg Br.

[6] Paternostro-Sluga T, Grim-Stieger M, Posch M, Schuhfried O, Vacariu G, Mittermaier C, Bittner C, Fialka-Moser V (2008). Reliability and validity of the Medical Research Council (MRC) scale and a modified scale for testing muscle strength in patients with radial palsy. J Rehabil Med.

[7] Kihlstrom C, Moller M, Lonn K, Wolf O (2017). Clavicle fractures: epidemiology, classification and treatment of 2 422 fractures in the Swedish Fracture Register; an observational study. BMC Musculoskelet Disord.

[8] Postacchini F, Gumina S, De Santis P, Albo F (2002). Epidemiology of clavicle fractures. J Shoulder Elbow Surg.

[9] Neer CS (1960). Nonunion of the clavicle. J Am Med Assoc.

[10] Rowe CR (1968). An atlas of anatomy and treatment of midclavicular fractures. Clin Orthop Relat Res.

[11] Altamimi SA, McKee MD, Canadian Orthopaedic Trauma S. Nonoperative treatment compared with plate fixation of displaced midshaft clavicular fractures. Surgical technique. J Bone Joint Surg Am. 2008;89(1):1-10. 10.2106/JBJS.G.01336 18310682

[12] Bhardwaj A, Sharma G, Patil A, Rahate V (2018). Comparison of plate osteosynthesis versus non-operative management for mid-shaft clavicle fractures-A prospective study. Injury.

[13] Thavarajah D, Scadden J (2013). Iatrogenic postoperative brachial plexus compression secondary to hypertrophic non-union of a clavicle fracture. Ann R Coll Surg Engl.

[14] Jeray KJ (2007). Acute midshaft clavicular fracture. J Am Acad Orthop Surg.

[15] Nordqvist A, Petersson CJ, Redlund-Johnell I (1998). Mid-clavicle fractures in adults: end result study after conservative treatment. J Orthop Trauma.

[16] Della Santa D, Narakas A, Bonnard C (1991). Late lesions of the brachial plexus after fracture of the clavicle. Ann Chir Main Memb Super.

